# 16S rRNA amplicon sequencing dataset for conventionalized and conventionally raised zebrafish larvae

**DOI:** 10.1016/j.dib.2016.06.057

**Published:** 2016-07-05

**Authors:** Daniel J. Davis, Elizabeth C. Bryda, Catherine H. Gillespie, Aaron C. Ericsson

**Affiliations:** a Department of Veterinary Pathobiology, University of Missouri, Columbia, MO 65201, USA; b University of Missouri Metagenomics Center (MUMC), University of Missouri, Columbia, MO 65201, USA

**Keywords:** Microbiome, Microbiota, Zebrafish larvae, 16S rRNA sequencing, Gnotobiotic

## Abstract

Data presented here contains metagenomic analysis regarding the sequential conventionalization of germ-free zebrafish embryos. Zebrafish embryos that underwent a germ-free sterilization process immediately after fertilization were promptly exposed to and raised to larval stage in conventional fish water. At 6 days postfertilization (dpf), these “conventionalized” larvae were compared to zebrafish larvae that were raised in conventional fish water never undergoing the initial sterilization process. Bacterial 16S rRNA amplicon sequencing was performed on DNA isolated from homogenates of the larvae revealing distinct microbiota variations between the two groups. The dataset described here is also related to the research article entitled “Microbial modulation of behavior and stress responses in zebrafish larvae” (Davis et al., 2016) [1].

## **Specifications Table**

**Table:** 

Subject area	*Biology*
More specific subject area	*Microbiome analysis in zebrafish larvae*
Type of data	*Table*
How data was acquired	*Illumina MiSeq*
Data format	*Raw, analyzed*
Experimental factors	*Reconstitution of sterilized embryos with conventional microbial populations*
Experimental features	1) *Microbial DNA extraction and amplification via PCR*
	2) *Bacterial 16S rRNA amplicon sequencing*
	3) *Trimming, filtering, and annotation of sequence data*
Data source location	*Columbia, MO, USA*
	*Latitude: 38.901366 Longitude: −92.2825 Altitude: 246 *m
Data accessibility	Data is within this article and available via http://www.ncbi.nlm.nih.gov/bioproject/321905

## **Value of the data**

• The data presented here can be used as justification for the use of zebrafish larvae as a model species in gnotobiotic research.
• These data are valuable in illustrating the consistency of microbial taxa present within a given group of larvae.
• These data will be of use in the selection of an appropriate methodology to generate gnotobiotic zebrafish larvae.

## 1 Data

Data presented here represent results of 16S rRNA sequencing of V4 region amplicons, generated using the Illumina MiSeq platform. Data are presented at the taxonomic levels of phylum, family, and operational taxonomic unit, and represent an average coverage of 4235 reads per sample (Table 1). This paper contains data related to the research concurrently published in Davis et al. [1].

## 2 Experimental design, materials and methods

### 2.1 Production of conventionalized and conventionally-raised zebrafish larvae

Wild-type zebrafish breeders were placed into a breeding tank overnight to spawn. Embryos were collected immediately after fertilization and evenly divided into separate groups for subsequent treatment. Conventionalized (CV) embryos were generated by following a previously published method [2]. Briefly, embryos were collected in sterile fish water containing 250 mg/mL amphotericin B, 5 µg/mL kanamycin, and 100 µg/mL ampicillin (AB-fish water). After sorting to remove unfertilized embryos, viable embryos were transferred to a tissue culture hood and gently washed 3 times in AB-fish water. Embryos were immersed in 0.1% PVP-Iodine solution for 2 min, and then immediately washed 3 times with sterile fish water. After washing, the embryos were immersed in 0.003% bleach solution for 1 h before being washed an additional 3 times with sterile fish water. Finally, the embryos were transferred into sterile tissue culture flasks containing conventional fish water. Conventionally raised (CR) embryos were transferred and maintained in conventional fish water immediately after collection without undergoing the sterilization process. All zebrafish embryos were maintained in a 28.5 °C incubator and raised at a density of ~1 embryo/mL until larval stage at 6 days postfertilization (dpf).

### 2.2 Microbial DNA extraction and quantification

Microbial DNA was extracted according to a modified previously published protocol [3]. Immediately following euthanasia, 12 zebrafish larvae were aseptically collected into 800 µL of lysis buffer (500 mM NaCl, 50 mM Tris–HCl, 50 mM EDTA, and 4% SDS), homogenized for 3 min in a Qiagen Tissuelyser II, and incubated at 70 °C for 20 min. Following centrifugation at 5000× g for 5 min at room temperature, the supernatant was mixed with 200 µL of 10 mM ammonium acetate, incubated on ice for 5 min, and then centrifuged at 16,000× g for 10 min at room temperature. 750 µL of supernatant was then mixed with an equal volume of chilled isopropanol, and incubated for 30 min on ice. The contents of the tube were then centrifuged at 16,000× g at 4 °C for 15 min to pellet DNA. The pellet was rinsed twice with 70% EtOH and re-suspended in 150 µL of tris-EDTA. 15 µL of proteinase-K and 200 µL of buffer AL (DNeasy kit, Qiagen, Valencia, CA) were then added and tubes were incubated at 70 °C for 10 min. 200 µL of 100% EtOH was then added and the entire contents of the tube were transferred to a Qiagen spin column before continuing with the manufacturer׳s instructions for DNA purification (DNeasy Kit, Qiagen). DNA was eluted in 50 µL of EB buffer (Qiagen). Yield of double-stranded DNA was determined via fluorometry (Qubit 2.0, Life Technologies, Carlsbad, CA) using Qubit® dsDNA BR assay kits (Life Technologies).

### 2.3 Metagenomic library preparation and sequencing

Sequencing of the V4 region of the 16S rRNA gene was performed on the Illumina MiSeq platform. Bacterial 16S rRNA amplicons were constructed by amplification of the V4 hypervariable region of the 16S rRNA gene with single-indexed primers flanked by Illumina standard adapter sequences. Universal primers (U515F/806R) previously developed against the V4 region were used for generating amplicons. Oligonucleotide sequences were obtained at proBase. A single forward primer and reverse primers with unique 12-base indices were used in all reactions. PCR reactions (50 µL) contained 100 ng of genomic DNA, forward and reverse primers (0.2 µM each), dNTPs (200 µM each), and Phusion High-Fidelity DNA Polymerase (1U). PCR amplification was performed as follows: amplification at 98 °C for 3 min, and 25 cycles at 98 °C for denaturation for 15 s, annealing at 50 °C for 30 s, and extension at 72 °C for 30 s, then a final extension at 72 °C for 7 min. Amplified product (5 µL) from each reaction was combined and thoroughly mixed; pooled amplicons were purified by addition of Axygen AxyPrep MagPCR Clean-up beads (50 µL) to an equal volume of 50 µL of amplicons and incubated at room temperature for 15 min. Products were washed multiple times with 80% EtOH and the dried pellet resuspended in Qiagen EB Buffer (32.5 µL), incubated at room temperature for 2 min, and then placed on a magnetic stand for 5 min. Supernatant (30 µL) was transferred to a low-binding microcentrifuge tube for storage. The final amplicon pool was evaluated using the Advanced Analytical Fragment Analyzer automated electrophoresis system, quantified with the Qubit flourometer using the quant-iT HS dsDNA reagent kit, and diluted according to the manufacturer׳s protocol.

### 2.4 Bioinformatics analysis

Assembly, binning, and annotation of DNA sequences were performed at the MU Informatics Research Core Facility (IRCF, Columbia, MO). Briefly, contiguous sequences of DNA were assembled using FLASH software [4] and contigs were culled if found to be short after trimming for a base quality less than 31. Qiime v1.7 [5] software was used to perform de novo and reference-based chimera detection and removal, and remaining contigs were assigned to operational taxonomic units (OTUs) using a criterion of 97% nucleotide identity. Taxonomy was assigned to selected OTUs using BLAST [6] against the Greengenes database [7] of 16S rRNA sequences and taxonomy.

## Figures and Tables

**Table 1:** Operational taxonomic units detected in 6 dpf conventionalized and conventionally-raised zebrafish larvae.

			**Conventionalized**			**Conventionally-raised**		
**Phylum**	**Family**	**Operational taxonomic unit**	Mean (%)	SEM (%)	Prevelance (%)	Mean (%)	SEM (%)	Prevelance (%)

		No blast hit;Other	2.69	0.39	100	2.22	0.19	100
*Actinobacteria*	*Microbacteriaceae*	*Family Microbacteriaceae*	0.00	0.00	0	0.00	0.00	25
	*Mycobacteriaceae*	*Mycobacterium sp.*	0.00	0.00	0	0.02	0.01	100
	*Bifidobacteriaceae*	*Bifidobacterium sp.*	0.02	0.02	25	0.00	0.00	0
	*unclassified*	*Order Solirubrobacterales*	1.82	0.19	100	1.08	0.05	100
*Bacteroidetes*	*unclassified*	*Order Bacteroidales*	0.46	0.16	100	0.13	0.03	100
	*Bacteroidaceae*	*Bacteroides sp.*	0.29	0.26	50	0.31	0.12	100
		*Bacteroides acidifaciens*	0.03	0.03	25	0.01	0.01	50
	*Porphyromonadaceae*	*Parabacteroides sp.*	0.25	0.22	50	0.04	0.02	75
	*Prevotellaceae*	*Family Prevotellaceae*	0.05	0.03	50	0.06	0.04	50
		*Prevotella sp.*	0.00	0.00	0	0.01	0.01	25
	*Rikenellaceae*	*Family Rikenellaceae*	0.15	0.06	75	0.02	0.01	75
	*S24-7*	*Family S24-7*	1.10	0.91	100	0.14	0.05	100
	*Barnesiellaceae*	*Family Barnesiellaceae*	0.02	0.02	25	0.01	0.00	50
	*Paraprevotellaceae*	*YRC22 sp.*	0.00	0.00	0	0.01	0.01	25
	*Cytophagaceae*	*Family Cytophagaceae*	0.07	0.04	50	5.05	0.09	100
		*Emticicia sp.*	0.00	0.00	0	0.13	0.02	100
		*Flectobacillus sp.*	0.00	0.00	0	3.19	0.13	100
		*Hymenobacter sp.*	0.00	0.00	0	0.72	0.05	100
		*Runella sp.*	0.00	0.00	0	2.22	0.12	100
		*Spirosoma sp.*	0.00	0.00	0	0.20	0.03	100
	*Cryomorphaceae*	*Fluviicola sp.*	0.00	0.00	0	0.05	0.01	100
	*Flavobacteriaceae*	*Flavobacterium sp.*	0.03	0.03	25	1.05	0.06	100
		*Flavobacterium columnare*	0.08	0.03	75	0.01	0.01	25
	*Weeksellaceae*	*Chryseobacterium sp.*	0.00	0.00	0	0.63	0.10	100
	*unclassified*	*Order Sphingobacteriales*	0.15	0.06	75	2.28	0.12	100
	*Sphingobacteriaceae*	*Pedobacter sp.*	0.21	0.07	100	1.20	0.09	100
		*Sphingobacterium multivorum*	0.00	0.00	0	17.50	0.53	100
	*Chitinophagaceae*	*Family Chitinophagaceae*	0.00	0.00	0	0.04	0.02	100
		*Sediminibacterium sp.*	0.06	0.03	50	0.01	0.01	25
	*Saprospiraceae*	*Family Saprospiraceae*	0.00	0.00	0	0.71	0.07	100
		*Saprospira sp.*	0.00	0.00	0	0.14	0.03	100
*Chloroflexi*	*SHA-31*	*Family SHA-31*	0.00	0.00	0	0.11	0.01	100
*Cyanobacteria*	*unclassified*	*Order YS2*	0.02	0.02	25	0.00	0.00	0
		*Order Stramenopiles*	12.56	1.22	100	8.96	0.49	100
*Deferribacteres*	*Deferribacteraceae*	*Mucispirillum schaedleri*	0.05	0.05	25	0.01	0.01	25
*Firmicutes*	*Staphylococcaceae*	*Staphylococcus succinus*	0.03	0.03	25	0.00	0.00	0
	*Lactobacillaceae*	*Lactobacillus sp.*	0.03	0.03	25	0.01	0.01	25
	*Turicibacteraceae*	*Turicibacter sp.*	0.00	0.00	0	0.00	0.00	25
	*unclassified*	*Order Clostridiales*	0.31	0.20	75	0.27	0.05	100
	*Clostridiaceae*	*Family Clostridiaceae*	0.02	0.02	25	0.00	0.00	0
		*Clostridium sp.*	0.10	0.10	25	0.04	0.02	50
	*Dehalobacteriaceae*	*Dehalobacterium sp.*	0.03	0.03	25	0.00	0.00	25
	*Lachnospiraceae*	*Family Lachnospiraceae*	0.10	0.04	75	0.07	0.01	100
		*Coprococcus sp.*	0.04	0.04	25	0.02	0.01	50
		*Coprococcus eutactus*	0.00	0.00	0	0.00	0.00	25
		*Roseburia sp.*	0.00	0.00	0	0.01	0.01	50
		*Ruminococcus gnavus*	0.00	0.00	0	0.00	0.00	25
	*Peptococcaceae*	*Family Peptococcaceae*	0.03	0.03	25	0.00	0.00	0
		*rc4-4 sp.*	0.02	0.02	25	0.00	0.00	25
	*Peptostreptococcaceae*	*Family Peptostreptococcaceae*	0.02	0.02	25	0.00	0.00	0
	*Ruminococcaceae*	*Family Ruminococcaceae*	0.18	0.07	75	0.19	0.04	100
		*Oscillospira sp.*	0.25	0.09	75	0.08	0.02	100
		*Ruminococcus sp.*	0.00	0.00	0	0.03	0.01	75
		*Ruminococcus flavefaciens*	0.05	0.03	50	0.00	0.00	0
	*Erysipelotrichaceae*	*Family Erysipelotrichaceae*	0.04	0.04	25	0.00	0.00	0
		*Allobaculum sp.*	0.09	0.09	25	0.00	0.00	25
*Proteobacteria*	*Caulobacteraceae*	*Family Caulobacteraceae*	1.15	0.25	100	0.14	0.01	100
		*Asticcacaulis sp.*	0.00	0.00	0	0.19	0.02	100
	*unclassified*	*Order RF32*	0.14	0.14	25	0.01	0.00	50
		*Order Rhizobiales*	0.02	0.02	25	0.02	0.00	100
	*Aurantimonadaceae*	*Family Aurantimonadaceae*	0.00	0.00	0	0.08	0.01	100
	*Bradyrhizobiaceae*	*Bosea genosp.*	0.03	0.03	25	0.01	0.01	50
	*Hyphomicrobiaceae*	*Hyphomicrobium sp.*	0.02	0.02	25	0.03	0.01	75
	*Phyllobacteriaceae*	*Family Phyllobacteriaceae*	0.03	0.03	25	0.04	0.01	100
	*Rhizobiaceae*	*Agrobacterium sp.*	0.09	0.06	50	0.05	0.01	100
	*Hyphomonadaceae*	*Family Hyphomonadaceae*	0.00	0.00	0	0.16	0.02	100
	*Rhodobacteraceae*	*Paracoccus aminovorans*	0.00	0.00	0	0.02	0.01	50
		*Rhodobacter sp.*	0.03	0.03	25	0.01	0.01	25
	*Rhodospirillaceae*	*Family Rhodospirillaceae*	0.00	0.00	0	0.11	0.01	100
		*Phaeospirillum fulvum*	0.00	0.00	0	0.07	0.02	100
	*unclassified*	*Order Rickettsiales*	1.00	0.14	100	0.73	0.10	100
	*Rickettsiaceae*	*Family Rickettsiaceae*	0.22	0.05	100	0.36	0.04	100
	*mitochondria*	*Vermamoeba vermiformis*	0.00	0.00	0	0.01	0.01	50
	*Sphingomonadaceae*	*Novosphingobium sp.*	0.00	0.00	0	0.00	0.00	25
		*Sphingomonas sp.*	0.30	0.06	100	0.06	0.03	100
		*Sphingomonas yabuuchiae*	0.00	0.00	0	0.16	0.04	100
		*Class Betaproteobacteria*	0.14	0.06	75	0.10	0.03	100
	*Alcaligenaceae*	*Sutterella sp.*	0.08	0.08	25	0.04	0.01	100
	*Comamonadaceae*	*Family Comamonadaceae*	2.21	0.19	100	22.21	0.38	100
		*Comamonas sp.*	0.05	0.03	50	0.15	0.02	100
		*Limnohabitans sp.*	1.77	0.42	100	6.53	0.08	100
		*Variovorax paradoxus*	0.13	0.05	75	0.07	0.03	100
	*Oxalobacteraceae*	*Family Oxalobacteraceae*	0.00	0.00	0	0.19	0.02	100
		*Janthinobacterium sp.*	0.00	0.00	0	0.01	0.01	25
	*Methylophilaceae*	*Methylotenera mobilis*	0.13	0.01	100	0.02	0.01	75
	*Rhodocyclaceae*	*Family Rhodocyclaceae*	4.47	0.26	100	3.16	0.13	100
	*Bdellovibrionaceae*	*Bdellovibrio sp.*	0.00	0.00	0	0.25	0.03	100
		*Bdellovibrio bacteriovorus*	1.59	0.13	100	0.43	0.03	100
	*unclassified*	*Order Myxococcales*	0.00	0.00	0	0.03	0.01	75
	*Helicobacteraceae*	*Family Helicobacteraceae*	0.03	0.03	25	0.00	0.00	0
	*Alteromonadaceae*	*Cellvibrio sp.*	0.00	0.00	0	2.83	0.18	100
	*Chromatiaceae*	*Rheinheimera sp.*	61.49	1.07	100	8.57	0.36	100
	*Coxiellaceae*	*Family Coxiellaceae*	0.00	0.00	0	0.01	0.01	50
	*Legionellaceae*	*Legionella sp.*	0.08	0.03	75	0.01	0.01	50
	*Moraxellaceae*	*Family Moraxellaceae*	0.00	0.00	0	0.03	0.01	100
	*Pseudomonadaceae*	*Pseudomonas sp.*	0.40	0.14	100	0.00	0.00	0
		*Pseudomonas pseudoalcaligenes*	0.00	0.00	0	0.04	0.00	100
	*Sinobacteraceae*	*Family Sinobacteraceae*	1.83	0.26	100	0.69	0.05	100
		*Nevskia ramosa*	0.83	0.17	100	2.71	0.12	100
	*Xanthomonadaceae*	*Stenotrophomonas sp.*	0.02	0.02	25	0.01	0.00	50
*Spirochaetes*	*Spirochaetaceae*	*Treponema sp.*	0.00	0.00	0	0.01	0.01	25
*Synergistetes*	*Dethiosulfovibrionaceae*	*Family Dethiosulfovibrionaceae*	0.00	0.00	0	0.01	0.01	25
*TM6*	*unclassified*	*Class SBRH58*	0.04	0.04	25	0.30	0.04	100
*TM7*	*F16*	*Family F16*	0.00	0.00	0	0.00	0.00	25
*Tenericutes*	*Anaeroplasmataceae*	*Anaeroplasma sp.*	0.00	0.00	0	0.00	0.00	25
	*unclassified*	*Order RF39*	0.00	0.00	0	0.02	0.00	100
*Verrucomicrobia*	*unclassified*	*Order HA64*	0.00	0.00	0	0.01	0.01	25
	*Opitutaceae*	*Family Opitutaceae*	0.00	0.00	0	0.09	0.02	100
	*RFP12*	*Family RFP12*	0.00	0.00	0	0.02	0.02	25
	*Verrucomicrobiaceae*	*Akkermansia muciniphila*	0.20	0.20	25	0.09	0.05	75
*Thermi*	*Deinococcaceae*	*Deinococcus sp.*	0.03	0.03	25	0.11	0.02	100

## References

[1] Davis D.J., Bryda E.C., Gillespie C.H., Ericsson A.C. (2016). Microbial modulation of behavior and stress responses in zebrafish larvae. Behav. Brain Res..

[2] Pham L.N., Kanther M., Semova I., Rawls J.F. (2008). Methods for generating and colonizing gnotobiotic zebrafish. Nat. Protoc..

[3] Hart M.L., Meyer A., Johnson P.J., Ericsson A.C. (2015). Comparative evaluation of DNA extraction methods from feces of multiple host species for downstream next-generation sequencing. PLoS One.

[4] Magoc T., Salzberg S.L. (2011). FLASH: fast length adjustment of short reads to improve genome assemblies. Bioinformatics.

[5] Kuczynski J., Stombaugh J., Walters W.A., Gonzalez A., Caporaso J.G., Knight R. (2012). Using QIIME to analyze 16S rRNA gene sequences from microbial communities. Curr. Protoc. Microbiol..

[6] Altschul S.F., Madden T.L., Schaffer A.A., Zhang J., Zhang Z., Miller W. (1997). Gapped BLAST and PSI-BLAST: a new generation of protein database search programs. Nucl. Acids Res..

[7] DeSantis T.Z., Hugenholtz P., Larsen N., Rojas M., Brodie E.L., Keller K. (2006). Greengenes, a chimera-checked 16S rRNA gene database and workbench compatible with ARB. Appl. Environ. Microbiol..

