# Supplementary material for: 16S rRNA amplicon sequencing dataset for conventionalized and conventionally raised zebrafish larvae
Source: Data Brief. 2016 Jul 5;8:938–43. doi: 10.1016/j.dib.2016.06.057 (PMC4961299; doi:10.1016/j.dib.2016.06.057)
Supplement: Supplementary file 1 — Supplementary material [file mmc1.docx]

**Conflict of interest**

The authors declare no competing financial interests or conflicts of interest.
